# Green nephrology: mitigating the environmental burden of dialysis and promoting sustainable kidney care

**DOI:** 10.3389/fmed.2026.1793544

**Published:** 2026-04-02

**Authors:** Yuxin Jiang, Shoulin Zhang

**Affiliations:** 1 College of Traditional Chinese Medicine, Changchun University of Chinese Medicine, Changchun, China; 2 Department of Nephrology, The Affiliated Hospital to Changchun University of Chinese Medicine, Changchun, China

**Keywords:** carbon footprint, climate change, eco-dialysis, environmental sustainability, green nephrology, hemodialysis

## Abstract

The relationship between kidney health and the environment is increasingly recognized as a bidirectional “vicious cycle.” As global temperatures rise due to climate change, the incidence of heat-stress nephropathy, acute kidney injury (AKI), and nephrolithiasis increases, thereby increasing demand for renal care. Paradoxically, the life-saving treatment for end-stage kidney disease (ESKD)—hemodialysis (HD)—is among the most resource-intensive interventions in modern medicine, with high water use, energy consumption, and waste generation. This Perspective article argues that nephrology must move from passive awareness to active mitigation. We summarize recent evidence showing that immediate clinical measures—such as reducing dialysate flow rates and adopting incremental HD—can lower environmental impact without compromising key treatment outcomes. We further argue that the sustainability hierarchy should begin upstream: delaying or avoiding dialysis through kidney-protective strategies, including appropriate dietary intervention, structured exercise, conservative kidney management, and transplantation where feasible. Finally, we emphasize that sustainable kidney care must also be patient-centered; interventions such as telemedicine are only truly sustainable when they are feasible, equitable, and acceptable for patients. Green nephrology should therefore integrate clinical optimization, engineering innovation, and patient-centered implementation into routine quality improvement.

## 1 Introduction: the hidden cost of saving lives

Hemodialysis (HD) is a technological marvel that sustains millions of lives worldwide. However, this success comes with a hidden environmental price tag. Contemporary estimates suggest that a standard HD treatment is highly resource intensive: depending on the system configuration, a single session may require roughly 300–500 L of water, and total water use may approach approximately 500 L when reverse osmosis (RO) rejection is included (1, 2). HD also consumes substantial electricity and relies heavily on single-use consumables and plastics (1, 2). For decades, this environmental burden was viewed as an unavoidable cost of doing business.

However, we can no longer ignore the broader context: the relationship between kidney care and the environment is bidirectional. As described by Goldfarb and Patel, the nephrology community is increasingly trapped in a “vicious cycle” in which healthcare emissions contribute to climate change, which in turn exacerbates kidney disease risks such as heat-stress nephropathy, acute kidney injury, and nephrolithiasis, thereby driving further demand for renal care (3). By failing to adopt greener practices, we may be inadvertently amplifying the very diseases we seek to treat.

Fortunately, the landscape is changing. A landmark 2024 multinational survey by the International Society of Nephrology demonstrated a substantial education gap: fewer than half of kidney care professionals reported adequate knowledge of the environmental impact of kidney care, and even fewer felt equipped to implement sustainable changes in routine practice (4). This gap between awareness and action is now one of the most pressing challenges in modern nephrology. We must equip clinicians with evidence-based, patient-centered strategies that reduce the environmental footprint of kidney care while preserving—or even improving—clinical quality.

## 2 The environmental footprint: quantified

To manage the problem, we must first measure it. The environmental footprint of kidney care is driven by three principal vectors: water consumption, waste generation, and energy intensity.

### 2.1 Water scarcity and inefficiency

Conventional HD is inherently inefficient in terms of water use. For every liter of ultrapure dialysate produced, conventional RO systems typically reject one to two liters of potable water down the drain (1, 2). Accordingly, a standard 4-h HD session may require approximately 300–500 L of water, with even greater total water demand once RO reject water is included (1, 2). In regions already experiencing water stress due to climate change, this level of consumption is becoming increasingly difficult to justify ethically and operationally.

### 2.2 The burden of waste

The proliferation of disposable technologies has worsened the waste trajectory of kidney care. Dialysis relies heavily on single-use plastics—tubing, dialyzers, saline bags, and packaging—primarily composed of polyvinyl chloride and polypropylene. A typical dialysis facility therefore generates large volumes of biomedical waste annually. Recent data from critical care nephrology further suggest that the waste burden is likely to worsen as extracorporeal therapies expand and the supply chain for consumables continues to dominate treatment-related emissions (5).

### 2.3 Carbon emissions

The carbon footprint of a dialysis patient is substantial. Early estimates suggested that the annual greenhouse gas emissions associated with treating a single HD patient may be comparable to driving a conventional internal-combustion vehicle for more than 40,000 kilometers (2). This footprint stems not only from direct electricity use by dialysis machines but also from water treatment losses, procurement of consumables, waste processing, and repeated patient travel to in-center facilities. As emphasized by the European Kidney Health Alliance, transport associated with thrice-weekly in-center HD is a major and often underappreciated source of emissions (6).

## 3 Strategies for a greener future

Moving beyond diagnosis of the problem, we present actionable strategies categorized into clinical optimization, engineering solutions, and care models (Table 1).

**Table 1:** Evidence-based strategies for reducing the environmental footprint of dialysis.

Intervention level	Strategy	Expected impact	Supporting evidence
Clinical optimization	Reduction of dialysate flow (QD)	Reduces water consumption by approximately 24 L/session without compromising Kt/V or middle-molecule removal	(7)
Clinical optimization	Incremental hemodialysis	Preserves residual kidney function and reduces resource use by starting with fewer sessions in selected patients	(8)
Clinical optimization	CKD-slowing dietary intervention and structured exercise	May delay dialysis initiation, reduce cumulative exposure to high-intensity KRT, and lower environmental impact through reduced red meat intake and better long-term functional status	(14–16)
Engineering	RO reject water recycling	Captures a substantial portion of total incoming water for non-clinical reuse	(1, 2)
Engineering	Renewable energy integration	Offsets electricity consumption of dialysis machines, HVAC systems, and water-treatment infrastructure	(5)
Care models	Context-sensitive modality planning (PD vs. HD)	Avoids oversimplified modality assumptions and supports locally optimized, lower-footprint care pathways	(9–11)
Care models	Telemedicine and remote monitoring	Reduces travel-related emissions and supports home-based pathways when implemented equitably	(6, 19)

### 3.1 Clinical optimization

#### 3.1.1 Optimized dialysate flow

Historically, clinicians often assumed that “more is better” with respect to dialysis intensity. However, emerging evidence challenges this dogma in favor of sustainability-conscious optimization. In a 2025 study, Ramos et al. showed that reducing dialysate flow from 500 to 400 mL/min in expanded hemodialysis saved approximately 24 L of water per treatment session without compromising Kt/V or middle-molecule removal (7). This is a particularly important finding because it offers a zero-cost, immediately implementable intervention that can reduce water use at scale without sacrificing treatment adequacy.

#### 3.1.2 Incremental hemodialysis

Incremental HD—starting with fewer than three sessions per week in appropriately selected patients with preserved residual kidney function—represents another clinically meaningful sustainability strategy. As reported by Aoun et al., this approach can preserve residual kidney function and reduce resource consumption linearly, with potential reductions of approximately 33–50% in water, energy, and consumables during the initial period of therapy (8). Importantly, such an approach is not a rationing strategy but rather an individualized prescription model grounded in physiology and patient selection.

### 3.2 Engineering innovations

#### 3.2.1 Renewable energy integration

Modern “green dialysis” centers are increasingly exploring integration of solar photovoltaic systems and other renewable energy sources to offset the energy demand of pumps, fluid warming, water treatment, lighting, and climate control. In parallel, energy-efficient equipment design is emerging as an important procurement criterion in critical care nephrology and dialysis infrastructure planning (5).

#### 3.2.2 Water recapture

RO reject water recapture is a clear example of a practical systems intervention. Although unsuitable for dialysate production, this water is generally of sufficient quality for non-clinical reuse, including laundry, toilet flushing, sanitation, and landscaping (1, 2). Recycling such water can meaningfully reduce the net freshwater footprint of dialysis units and move facilities away from a linear “take-make-waste” model.

### 3.3 Sustainable care models

Peritoneal dialysis (PD) is often proposed as a lower-impact alternative to in-center HD, but the magnitude—and even the direction—of this environmental advantage is context dependent. Contemporary lifecycle assessments show that in-center HD is frequently dominated by electricity use, water treatment losses, and patient transport, whereas PD shifts a greater proportion of environmental burden upstream to consumables manufacturing, packaging, and supply-chain logistics (9–11). Importantly, these studies also show that “modality” is not a single exposure: the carbon intensity of the local electricity grid, travel distances, treatment frequency, and procurement pathways can substantially alter the relative footprint of PD versus HD (9–11). Accordingly, sustainable modality planning should be framed as a locally optimized systems decision rather than as a universal hierarchy.

### 3.4 A sustainability hierarchy: dialysis avoidance, transplantation, and modality transitions

While this manuscript focuses on reducing the environmental burden of HD, the most effective way to decrease dialysis-associated emissions is to prevent, delay, or avoid the need for dialysis altogether. A systems perspective across the chronic kidney disease (CKD) trajectory suggests that upstream interventions—slowing progression, optimizing conservative kidney management where appropriate, and improving timely referral and preparation—can yield the largest absolute reductions in resource use because they reduce cumulative exposure to high-intensity kidney replacement therapy (KRT) (12, 13). In other words, “green dialysis” should be situated within an integrated kidney-care framework that prioritizes delaying CKD stage 5 whenever clinically feasible.

In this context, dietary intervention deserves explicit attention. Appropriately supervised low-protein, plant-forward dietary patterns may help delay CKD progression, reduce metabolic burden, and lower the environmental footprint of care by reducing reliance on red meat and other high-emission foods (14). Such strategies must be individualized to avoid protein-energy wasting, hyperkalemia, or inadequate caloric intake, especially in advanced CKD or frail patients. Nonetheless, from both a nephrology and sustainability perspective, dietary counseling should be regarded as an upstream intervention rather than a peripheral lifestyle add-on (14).

Structured physical exercise should also be considered part of this upstream sustainability strategy. Exercise-based renal rehabilitation improves physical function and may support longer-term kidney-preserving care pathways. In the AVANTE-HEMO study, oral nutritional supplementation combined with exercise improved physical-function outcomes in adult HD patients, supporting the feasibility and clinical value of pairing nutritional and exercise interventions in kidney care (15). In addition, Piva et al. reported that a home-based exercise program in older patients with peripheral artery disease and CKD was associated with more favorable long-term renal trends and fewer dialysis initiations than usual care, although these findings require confirmation in randomized studies (16). Taken together, these data support the Reviewer’s important point: lifestyle-based interventions may simultaneously improve patient outcomes and reduce cumulative dependence on resource-intensive KRT.

Kidney transplantation is also central to an environmentally coherent KRT strategy. Comparative lifecycle assessments of KRT options consistently identify transplantation as the lowest-impact modality over time relative to chronic dialysis, although the total footprint still depends on surgical pathways, immunosuppression supply chains, and follow-up intensity (10). At the same time, access to transplantation remains profoundly unequal worldwide; therefore, discussions of sustainability must explicitly acknowledge that expanding equitable transplant capacity can be both clinically and environmentally beneficial, but requires system-level investment and governance (17).

Finally, sustainability comparisons between PD and HD must incorporate real-world modality transitions. PD technique failure—particularly ultrafiltration failure—often necessitates transfer to HD, and thus any “PD-first” environmental strategy should be paired with planned pathways for safe transition, contingency capacity, and patient-centered shared decision-making (18). Environmental analyses should therefore reflect intended modality choice as well as expected rates of switching.

In parallel, telemedicine and remote patient monitoring can function as cross-cutting decarbonization tools by reducing patient travel, enabling earlier troubleshooting, and supporting home-based care pathways (6, 19). However, sustainability must be understood not only environmentally, but also in terms of patient feasibility. Not all patients have sufficient technological literacy, reliable internet access, caregiver support, or confidence with digital tools. As emphasized in recent telenephrology literature, technological difficulty, low eHealth literacy, and the “digital divide”—particularly among older adults—are major barriers to implementation (19). Therefore, telemedicine should be framed as a complement to, rather than a replacement for, face-to-face care, and its implementation should include equity safeguards such as device access, patient training, caregiver engagement, and hybrid care pathways (19).

## 4 Challenges and policy recommendations

Decarbonizing dialysis cannot be approached as a single global blueprint because HD practice and enabling infrastructure vary substantially across countries and regions. International data describing the organization, financing, and operational realities of HD services underscore marked heterogeneity inwater-treatment capacity, staffing, supply-chain reliability, and oversight mechanisms (20). At the same time, broader global analyses of kidney disease burden and service access highlight entrenched inequities that shape which sustainability interventions are feasible in a given setting—for example, local energy mix, water scarcity, waste-management capacity, reimbursement structures, and access to trained staff (21). Consequently, “green” recommendations should be framed as a menu of locally adaptable levers, prioritized by regional constraints and co-benefits for patient safety and access.

Importantly, sustainability should not be interpreted solely in environmental terms. A strategy is not truly sustainable if patients cannot realistically adopt or maintain it. Low-flow HD, incremental schedules, home therapies, telemedicine, dietary modification, and exercise programs all require patient understanding, support, and local feasibility. Older age, frailty, limited digital literacy, food insecurity, low health literacy, and lack of caregiver support may constrain implementation. Sustainable kidney care must therefore remain explicitly patient-centered and equity-sensitive, rather than being reduced to a purely technical emissions-reduction exercise (19–21).

Achieving measurable reductions in the carbon footprint of HD requires coordinated action from multiple stakeholders (Table 2). Beyond clinicians and dialysis providers, influential actors include payers and purchasers (through reimbursement design and tender specifications), regulators (through safety-aligned enabling policies for water recapture and selective reuse), manufacturers (through redesign of consumables and transparent product lifecycle assessments), facility engineers and utilities (through water and energy infrastructure), and patient organizations (through shared decision-making and acceptability of care-model change) (6, 20).

**Table 2:** Key stakeholders and high-leverage actions for sustainable kidney care.

Stakeholder	Primary levers	Examples of actionable steps
Clinicians and nurses	Prescription and care-model decisions	Implement dialysate flow reduction protocols; select incremental HD for eligible patients; incorporate environmental impact into shared decision-making; refer appropriate patients for dietary counseling, exercise-based renal rehabilitation, conservative kidney management, home therapies, and transplant evaluation
Patients and caregivers	Choice, adherence, and logistics	Participate in shared decision-making about modality and visit structure; optimize transport planning where possible; engage in feasible dietary and exercise interventions; participate in safe home waste segregation where regulations allow
Biomedical engineers and facility managers	Water and energy infrastructure	Optimize RO system efficiency and recapture reject water; conduct preventive maintenance to reduce leaks and inefficiency; perform energy audits and develop climate-resilient backup systems
Hospital and dialysis administrators	Governance and procurement	Mandate environmental KPIs; enforce green tenders and vendor scorecards; support hybrid care pathways, staff training, and data systems for environmental tracking
Industry (manufacturers and suppliers)	Design and supply chain	Develop lower-carbon materials and machines; design recyclable packaging and take-back programs; publish transparent lifecycle assessments for products
Waste contractors	Waste processing pathways	Improve on-site segregation training; explore sterilization and recycling options for non-infectious plastics; track and report waste stream volumes
Policymakers and regulators	Incentives, standards, and equity	Establish accreditation standards for sustainability; reimburse telehealth and home therapies where appropriate; review regulations to enable safe water recycling and reuse; build equity safeguards so environmentally favorable interventions remain accessible to patients with lower digital literacy or fewer resources
Professional societies	Guidelines and training	Integrate sustainable nephrology into medical curricula; publish clinical guidance for green kidney care; establish multicenter benchmarking networks for environmental data

Embedding environmental key performance indicators (KPIs) into routine quality reporting and procurement contracts can operationalize this accountability and reduce variability in implementation across settings. As noted by Arias-Guillén et al., nephrology still lacks universally adopted environmental KPIs for dialysis (1). We therefore propose that providers begin by reporting at least “Water per Session” and “Energy per Session” alongside conventional outcomes such as mortality, hospitalization, and treatment adequacy. Hospital administrators should also integrate environmental criteria into tenders for dialysis machines and consumables, favoring vendors with transparent decarbonization roadmaps. Finally, regulators should revisit restrictions on safe reuse and water recycling where standards can be met without compromising infection control or patient safety.

**Figure 1:**
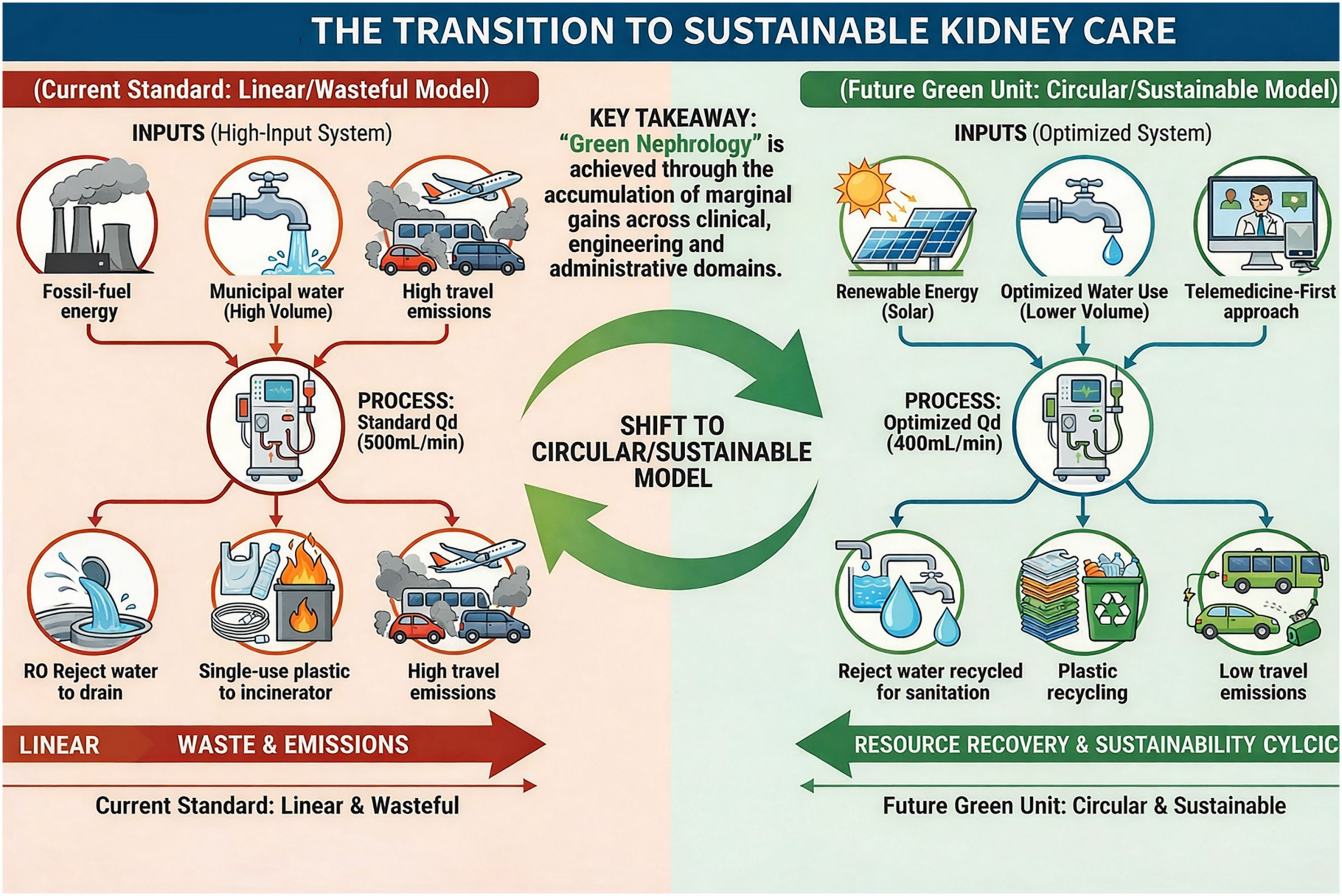
The transition to sustainable kidney care.

Figure 1 summarizes the proposed transition from resource-intensive dialysis-centered care toward a broader, patient-centered sustainable kidney-care framework.

## 5 Conclusion

Sustainability in kidney care is no longer a niche concern; it is a clinical quality issue. The vicious cycle linking climate change and kidney disease demands an urgent response. Recent evidence shows that practical clinical adjustments, such as reduced dialysate flow and incremental HD, can lower environmental impact without compromising treatment effectiveness (7, 8). At the same time, the most powerful sustainability strategy is upstream: delaying or avoiding dialysis through kidney-protective care, including dietary intervention, exercise, conservative management, and timely transplantation where feasible.

Equally important, sustainable kidney care must remain patient-centered. An intervention cannot be called sustainable if it is environmentally attractive but infeasible for the people expected to use it. The future of green nephrology therefore lies in integrating environmental accountability with clinical judgment, equity, and real-world patient feasibility.

We call upon the global nephrology community to act. For researchers, the priority is to generate robust lifecycle assessment and implementation data. For clinicians, the task is to adopt individualized, evidence-based low-impact care pathways where appropriate. For policymakers, the duty is to mandate measurement, support equitable implementation, and align reimbursement with sustainability goals. Nephrologists have long led the way in life-sustaining technology; it is now time to lead the way in life-sustaining policy—for our patients and for our planet.

## Data Availability

The original contributions presented in the study are included in the article/supplementary material, further inquiries can be directed to the corresponding author.

## References

[1] Arias-Guillén M Martínez Cadenas R Gómez M Martín Vaquero N Pereda G Audije-Gil J . Environmental challenges in hemodialysis: exploring the road to sustainability. Nefrologia. (2024) 44:784–95. doi: 10.1016/j.nefroe.2024.11.021, 39645513

[2] Agar JWM. Green dialysis: the environmental challenges ahead. Semin Dial. (2015) 28:186–92. doi: 10.1111/sdi.12324, 25440109

[3] Goldfarb DS Patel AA. Climate change and its implications for kidney health. Curr Opin Urol. (2024) 34:377–83. doi: 10.1097/MOU.0000000000001197, 38881301

[4] Sandal S Ethier I Onu U Fung W Bajpai D Bilchut WH . Climate change, kidney health, and environmentally sustainable kidney care: a multinational survey of health care professionals. Clin J Am Soc Nephrol. (2024) 35:1084–94. doi: 10.1681/ASN.0000000000000402, 38768364 PMC11377800

[5] Molano-Triviño A Rizo-Topete LM Zúñiga E Castellanos-De la Hoz JC Karopadi AN. Critical care nephrology: opportunities for implementing green practices. Front Med. (2025) 12:1635718. doi: 10.3389/fmed.2025.1635718, 41103690 PMC12521179

[6] Vanholder R Agar J Braks M Gallego D Gerritsen KGF Harber M . The European green Deal and nephrology: a call for action by the European kidney health Alliance. Nephrol Dial Transplant. (2023) 38:1080–8. doi: 10.1093/ndt/gfac160, 35481547

[7] Ramos C Gutiérrez JF Sanabria RM Vesga J la Castelnos De Hoz J Zuniga-Rodriguez E . Toward green dialysis: efficacy and sustainability with reduced dialysate flow in expanded hemodialysis. Blood Purif. (2026) 55:148–55. doi: 10.1159/000548892 41166524 PMC12707869

[8] Aoun M Finianos S Beaini C Sleilaty G Ghaleb R Nourie N . Twice against thrice-weekly hemodialysis (TATH): a multicenter nonrandomized trial. BMC Nephrol. (2025) 26:176. doi: 10.1186/s12882-025-04105-3, 40188011 PMC11972488

[9] Barraclough KA Talbot B Knight J Blair S McGain F Masterson R . Carbon emissions from different dialysis modalities: a life cycle assessment. Am J Kidney Dis. (2025) 86:465–474.e1. doi: 10.1053/j.ajkd.2025.04.019, 40602678

[10] Saleem S Stigant C Rajan T Hewage K Sadiq R MacNeill AJ . Environmental impacts of kidney replacement therapies: a comparative lifecycle assessment. Am J Kidney Dis. (2026) 87:65–74.e1. doi: 10.1053/j.ajkd.2025.08.010, 41076003

[11] Makhloufi M Cottinet PJ Ranchin B Dureuil B Loppinet T Grinberg D . Haemodialysis versus peritoneal dialysis in children: an eco-audit. Nephrol Dial Transplant. (2024) 39:1927–9. doi: 10.1093/ndt/gfae159, 39003241 PMC11522548

[12] Barraclough KA Cases A Eckelman MJ Germond-Duret C Zoccali C Embleton N . International environmental impact of CKD care. Kidney Int Rep. (2026) 11:103662. doi: 10.1016/j.ekir.2025.10.019, 41550528 PMC12805029

[13] Hole B Wearne N Arruebo S Caskey FJ Damster S Donner JA . Global access and quality of conservative kidney management. Nephrol Dial Transplant. (2024) 39:ii35–42. doi: 10.1093/ndt/gfae129, 39235199

[14] Carrero JJ González-Ortiz A Avesani CM Bakker SJL Bellizzi V Chauveau P . Plant-based diets to manage the risks and complications of chronic kidney disease. Nat Rev Nephrol. (2020) 16:525–42. doi: 10.1038/s41581-020-0297-2, 32528189

[15] Martin-Alemañy G Espinosa-Cuevas MÁ Pérez-Navarro M Wilund KR Miranda-Alatriste P Cortés-Pérez M . Effect of oral nutritional supplementation with and without exercise on nutritional status and physical function of adult hemodialysis patients: a parallel controlled clinical trial (AVANTE-HEMO study). J Ren Nutr. (2020) 30:126–36. doi: 10.1053/j.jrn.2019.06.010 31607547

[16] Piva G Crepaldi A Lamberti N Caruso L Rinaldo N Manfredini R . Home-based exercise in elderly patients with claudication and chronic kidney disease is associated with lower progressive renal function worsening: a 5-year retrospective study. Meta. (2023) 13:56. doi: 10.3390/metabo13010056 PMC9862132 36676981

[17] Viecelli AK Gately R Barday Z Shojai S Arruebo S Caskey FJ . Worldwide organization and structures for kidney transplantation services. Nephrol Dial Transplant. (2024) 39:ii26–34. doi: 10.1093/ndt/gfae144, 39235196

[18] Teitelbaum I. Ultrafiltration failure in peritoneal dialysis: a pathophysiologic approach. Blood Purif. (2015) 39:70–3. doi: 10.1159/000368972, 25661912

[19] Sugawara Y Hirakawa Y Nangaku M. Telemedicine in nephrology: future perspective and solutions. Clin Kidney J. (2024) 17:ii1–8. doi: 10.1093/ckj/sfae267, 39583140 PMC11581765

[20] Htay H Cho Y Jha V See E Arruebo S Caskey FJ . Global structures, practices, and tools for provision of hemodialysis. Nephrol Dial Transplant. (2024) 39:ii11–7. doi: 10.1093/ndt/gfae131, 39235197

[21] Bello AK Okpechi IG Levin A Ye F Damster S Arruebo S . An update on the global disparities in kidney disease burden and care across world countries and regions. Lancet Glob Health. (2024) 12:e382–95. doi: 10.1016/S2214-109X(23)00570-3, 38365413

